# *In Vitro* Dissolution Methods for Hydrophilic and Hydrophobic Porous Silicon Microparticles

**DOI:** 10.3390/pharmaceutics3020315

**Published:** 2011-06-21

**Authors:** Juha Mönkäre, Joakim Riikonen, Elina Rauma, Jarno Salonen, Vesa-Pekka Lehto, Kristiina Järvinen

**Affiliations:** 1 School of Pharmacy, Pharmaceutical Technology, Faculty of Health Sciences, University of Eastern Finland, 70211 Kuopio, Finland; E-Mails: elina.rauma@apteekit.net (E.R.); kristiina.jarvinen@uef.fi (K.J.); 2 Department of Applied Physics, Faculty of Science and Forestry, University of Eastern Finland, 70211 Kuopio, Finland; E-Mails: joakim.riikonen@uef.fi (J.R.); vesa-pekka.lehto@uef.fi (V.-P.L.); 3 Department of Physics and Astronomy, University of Turku, 20014 Turku, Finland; E-Mail: jarno.salonen@utu.fi (J.S.)

**Keywords:** porous silicon, microparticles, drug release, *in vitro* release methods, peptide

## Abstract

Porous silicon (PSi) is an innovative inorganic material that has been recently developed for various drug delivery systems. For example, hydrophilic and hydrophobic PSi microparticles have been utilized to improve the dissolution rate of poorly soluble drugs and to sustain peptide delivery. Previously, the well-plate method has been demonstrated to be a suitable *in vitro* dissolution method for hydrophilic PSi particles but it was not applicable to poorly wetting hydrophobic thermally hydrocarbonized PSi (THCPSi) particles. In this work, three different *in vitro* dissolution techniques, namely centrifuge, USP Apparatus 1 (basket) and well-plate methods were compared by using hydrophilic thermally carbonized PSi (TCPSi) microparticles loaded with poorly soluble ibuprofen or freely soluble antipyrine. All the methods showed a fast and complete or nearly complete release of both model compounds from the TCPSi microparticles indicating that all methods described *in vitro* dissolution equally. Based on these results, the centrifuge method was chosen to study the release of a peptide (ghrelin antagonist) from the THCPSi microparticles since it requires small sample amounts and achieves good particle suspendability. Sustained peptide release from the THCPSi microparticles was observed, which is in agreement with an earlier *in vivo* study. In conclusion, the centrifuge method was demonstrated to be a suitable tool for the evaluation of drug release from hydrophobic THCPSi particles, and the sustained peptide release from THCPSi microparticles was detected.

## 1. Introduction

Porous silicon (PSi) is an inorganic mesoporous material that has been developed in recent years for particulate drug delivery systems and it is fabricated from silicon wafers by electrochemical etching of silicon in hydrofluoric acid [1,2]. The native PSi surface is chemically unstable and is therefore typically stabilized by different surface treatments which not only increase the surface stability, but also modify the hydrophobicity or hydrophilicity of the surface [1]. The drug is most often loaded into the pores of the PSi particles by an immersion technique and the advantages of the loading process are that it allows mild conditions which do not cause drug degradation, and it can achieve high loading degrees that can be as high as 40–50% w/w [3-6]. In oral drug delivery research, PSi microparticles have enhanced the *in vitro* dissolution of poorly soluble compounds [3-5] as well as drug permeation through Caco-2 monolayers [5,7]. Recently, it was shown that thermally hydrocarbonized PSi (THCPSi) microparticles could sustain *in vivo* delivery of peptides involved in the regulation of food intake [8,9].

Unlike the situation for tablets or capsules, there is no gold standard for assessing release from particulate drug delivery systems, instead, many different *in vitro* drug release tests have been developed and the field is still evolving. The commonly used methods for testing of drug release from particulate drug delivery systems can be divided into three classes: 1) sample and separate; 2) dialysis; and 3) continuous flow methods [10,11]. Earlier, well-plates with cell culture inserts have been commonly used to study drug release from hydrophilic thermally oxidized (TOPSi) or thermally carbonized (TCPSi) PSi microparticles [3-5]. However, the well-plate method is not suitable for hydrophobic THCPSi microparticles as the wettability of these particles is poor and therefore, they are not homogenously dispersed in the dissolution medium but float on the surface [12].

The objective of this work was to compare three *in vitro* dissolution methods, namely centrifuge, USP Apparatus 1 (basket) and well-plate method, for testing of drug release from hydrophilic porous silicon microparticles. Based on these results, an *in vitro* dissolution method was developed for evaluating peptide (ghrelin antagonist, GhA) release from hydrophobic porous silicon microparticles.

## 2. Experimental Section

### 2.1. Materials

Silicon wafers Si (100) of p+-type with resistivity values of 0.01–0.02 Ωcm (Cemat silicon, Poland) were used in the preparation of PSi. Ibuprofen was acquired from Orion Pharma (Espoo, Finland), antipyrine, was purchased from Sigma-Aldrich (St. Louis, Missouri, USA) and ghrelin antagonist ([D-Lys-3]-GHRP-6, H-His-D-Trp-D-Lys-Trp-D-Phe-Lys-NH_2_) was purchased from Peptides International Inc. (Louisville, Kentucky, USA). Deionized water was processed by the Milli-Q system (Gradient AS-10, Millipore). Solvents used in HPLC analysis were HPLC grade and reagents used in making the phosphate-buffered saline (PBS, pH 7.4) were reagent grade. Ethanol (99.5%) used in electrolyte and loading solution was purchased from Altia (Finland) and methanol and HF (37%–39%) from Merck KGaA (Germany).

### 2.2. Preparation and characterization of TCPSi and THCPSi microparticles

The PSi was prepared by anodizing the wafers in an HF (38%)–ethanol mixture (HF:EtOH, 1:1) with a current density of 50 mA/cm^2^. The process was performed in the dark and free-standing films were obtained by abruptly increasing the current. Free-standing porous silicon films were ball-milled after anodization, followed by sieving to obtain a particle size < 38μm. Thermal carbonization and thermal hydrocarbonization were used to stabilize the PSi surface after the oxidized surface had been replaced by the hydrogen-terminated surface. The surface modification followed the procedure described by Salonen *et al.* [13,14] and Limnell *et al.* 2007 [12]. The surfaces of thermally carbonized PSi (TCPSi) and thermally hydrocarbonized PSi (THCPSi) are hydrophilic and hydrophobic, respectively, and they are chemically more stable than the untreated as-anodized surface [13,14].

The drug loading was performed in ethanol or in water for ibuprofen and antipyrine, respectively. The particles were soaked either in 300 mg/mL ibuprofen solution for one hour or in 1.1 g/mL antipyrine solution for 1.5 h. Subsequently, loaded microparticles were vacuum filtrated from the solution and finally, the loaded microparticles were dried in an oven at 65 °C for 1 h. Two batches of TCPSi particles were loaded with ibuprofen. One batch was used in the centrifuge and well-plate method and with the other being examined in the USP Apparatus 1 (basket) method. The pore volumes of the batches (2.1 and 1.7 cm^3^/g) were different, leading to some discrepancies also in the loading degrees.

GhA was dissolved in methanol and mesoporous THCPSi microparticles were soaked in the peptide solution for 1.5 h at room temperature. The loading solution was subjected to ultrasound 3 times during loading to ensure homogenous loading. The particles were filtered from the solution and dried for 4 h at room temperature.

The loading degree of samples (m_drug_/(m_particles_ + m_drug_) and the amount of crystallized substance on the external surface of the microparticles (m_drug on surface_ /(m_particles_ + m_drug_) were characterized with thermogravimetry (TG; TGA 7, Perkin Elmer, 10 °C/min, N2 gas purge) and differential scanning calorimetry (DSC; Pyris Diamond DSC, Perkin Elmer, 10 °C/min, N_2_ gas purge) as described earlier [15].

### 2.3. Drug release experiments

#### 2.3.1. Centrifuge method

Ibuprofen, antipyrine and GhA loaded microparticles (1 mg) were weighed into the microcentrifuge tubes and suspended in 1.5 mL of pH 7.4 PBS at +37 °C. In the case of GhA release, 0.1% w/V bovine serum albumin (BSA) was added to the PBS in order to prevent peptide adsorption to lab ware. Sink conditions were maintained: the maximum theoretical concentrations of dissolved ibuprofen, antipyrine and GhA were 20%, < 0.1% and < 1% of their saturation concentrations, respectively. The microcentrifuge tubes were placed in the water bath shaker with orbital shaking at a frequency of 120 strokes/min at +37 °C (Grant OLS200, Cambridge, UK). At pre-determined time intervals, the tubes were centrifuged for 2 minutes (13 000 rpm, 17 000 g, Heraues Biofuge Fresco, Osterode, Germany) and the supernatant was collected for the analysis of the drug concentration. The microparticles were re-suspended in the fresh buffer before the tubes were replaced in the shaker.

#### 2.3.2. USP Apparatus 1 (basket)

Ibuprofen release from the TCPSi microparticles was studied by the USP Apparatus 1 (basket) (Sotax AT6, Sotax AG, Basel, Switzerland) method. Ibuprofen-loaded TCPSi microparticles (40 mg) were weighed into soft gelatin capsules (size 00). The volume of pH 7.4 PBS was 900 mL (+37 °C) and the rotation speed of the basket was 100 rpm. Sink conditions were maintained for ibuprofen: the maximum theoretical concentration of dissolved ibuprofen was < 1 % of the saturation concentration. A sample of 5 mL was withdrawn at pre-determined time intervals and this volume was replaced by fresh buffer. The samples were filtered through a 0.45 μm Minisart filter (Sartorius, Goettingen, Germany) before the analysis of the drug concentration.

#### 2.3.3. Well-plate method

The well-plate method was adapted from the earlier study of Salonen *et al.* [3]. Transwell cell culture inserts were used as the donor chamber and 6-well culture plates (Corning Corp., Corning NY, USA) as the acceptor chamber. Two different membrane materials, polyester and polycarbonate membranes (Transwell, Corning Corp., Corning, NY, USA) separating the chambers were studied in order to evaluate the effect of the membrane on dissolution. Both membranes had an identical membrane area (4.7 cm^2^) and pore size (0.4 μm). However, the pore densities of the membranes were different, as the pore density of the polycarbonate membrane (1 × 10^8^ pores/cm^2^) was 25 times higher than that of the polyester membrane (4 × 10^6^ pores/cm^2^). In order to clarify the effects of the studied membranes on the ibuprofen and antipyrine release rates from the microparticles, the transport of the drugs, applied either as a solution or powder to the donor chamber, across the membranes was also determined.

Plain drug powder (1 mg) or drug-loaded TCPSi microparticles (2 mg) was weighed to the donor chamber and 1.5 mL of pH 7.4 PBS was added to the donor chamber on top of the samples, and 2.75 mL was pipette into the acceptor chamber. In the case of the drug solution, 1.5 mL of 0.67 mg/mL drug solution in pH 7.4 PBS (*i.e.* 1 mg of drug) was added to the donor chamber. Sink conditions were maintained for antipyrine: the maximum theoretical concentration of dissolved antipyrine in donor chamber was < 0.1% of the saturation concentration. Non-sink conditions were utilized for ibuprofen: the maximum theoretical concentrations of dissolved ibuprofen in donor chamber were 37% and 48% of the saturation concentration for microparticles and powder, respectively. Cell culture plates were placed in a temperature-controlled (+37 °C) orbital shaker with constant shaking at 130 rpm (Titramax 1000 and Heidolph Inkubator 1000, Heidolph Instruments, Germany). At pre-determined time intervals, donor chambers were moved onto the top of the next acceptor chamber with fresh pH 7.4 PBS buffer, and the sample was collected from the previous acceptor chamber.

### 2.4. Drug analysis

In the tests with the centrifuge and well plate methods, ibuprofen (λ = 220 nm) and antipyrine (λ = 240 nm) concentrations in the samples were analyzed with a UV-spectrophotometer (Thermo Spectronic Genesys 10, Madison WI, USA). In the tests with the USP Apparatus 1 (basket), ibuprofen concentrations were analyzed in a Gilson High Performance Liquid Chromatograph (HPLC). The system consisted of UV detector (UV/VIS-151), pump (321), autoinjector (234), interface (506C) and integrator (Unipoint 3.0). The mobile phase was a mixture of acetonitrile (70% v/v), water (30% v/v) and trifluoroacetic acid (0.1% v/v). The analytical column was a reverse-phase Supelcosil® C-8 column (150 × 4.6 mm id, particle size 5 μm, Supelco, Bellefonte, PA, USA). The injection volume was 20 μL, flow rate 1 mL/min, and ibuprofen was detected at 214 nm. GhA was analyzed with a similar HPLC system as used for ibuprofen but the interface unit was Hercule Lite for Borwin 1.5 and the integrator was a Borwin 1.5. The mobile phase was a mixture of acetonitrile (73% v/v), water (27% v/v) and trifluoroacetic acid (0.1% v/v). The analytical column was a reverse-phase Supelcosil® LC-18-DB column (150 × 4.6 mm id, particle size 5 μm, Supelco, Bellefonte, PA, USA). The injection volume was 100 μL, flow rate 1 mL/min, and ibuprofen was detected at 220 nm.

### 2.5. Statistical analysis

The non-parametric Kruskal-Wallis test (SPSS 14.0 for Windows) was used to test the statistical significance of differences between methods. The post hoc test [16] was employed to test the significance of the differences of the means. The level of significance was taken as p < 0.05.

## 3. Results and Discussion

### 3.1. Comparison of the in vitro dissolution methods

USP Apparatus 1 (basket), centrifuge and well plate methods were compared by using hydrophilic TCPSi microparticles loaded with a poorly soluble ibuprofen and freely soluble antipyrine (Table 1). In the present study, the solubility of ibuprofen at pH 7.4 PBS (+37 °C) was determined to be 1.68 mg/mL whereas antipyrine is freely soluble in water (>1000 mg/mL) [3]. High drug loading degrees were achieved for antipyrine loaded microparticles (56% w/w) and two batches of ibuprofen loaded microparticles (47% w/w for centrifuge and well-plate methods, 31% w/w for USP Apparatus 1). In addition, virtually no crystallized (<2% w/w) drug was observed on the external surfaces of the studied microparticles. The differences in the loading degrees of ibuprofen are due to different pore volumes of the particles, as a correlation between pore volumes and drug loading degrees has been recently demonstrated [6].

**Figure 1.**
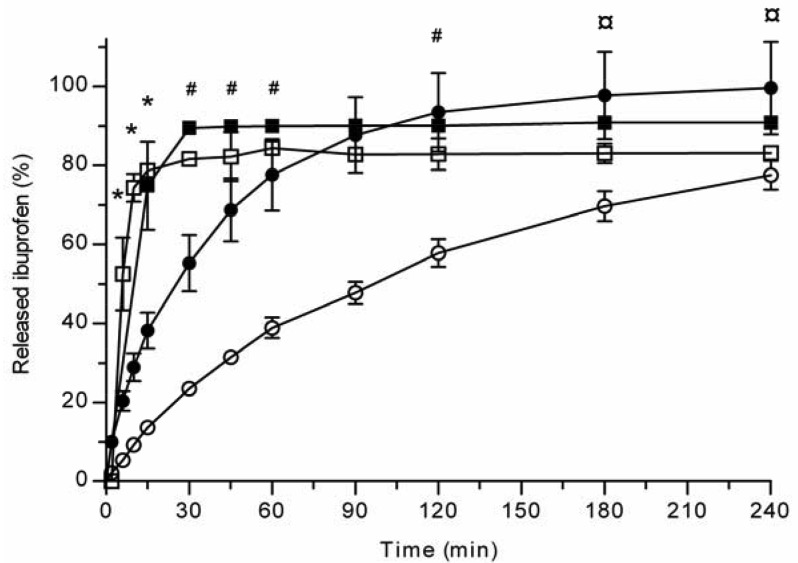
Ibuprofen release (%) from TCPSi microparticles at pH 7.4 PBS (+37 °C) with centrifuge (■), USP Apparatus 1 (basket) (□) and well-plate methods (polyester (○) and polycarbonate (•) membranes). Mean ± SD values are shown (n = 3), Statistical differences: *p < 0.05 USP Apparatus 1 *vs.* well-plate (polyester membrane), # p < 0.05 centrifuge vs. well-plate (polyester membrane), ¤ p < 0.05 well-plate (polycarbonate membrane) *vs.* well-plate (polyester membrane).

**Figure 2.**
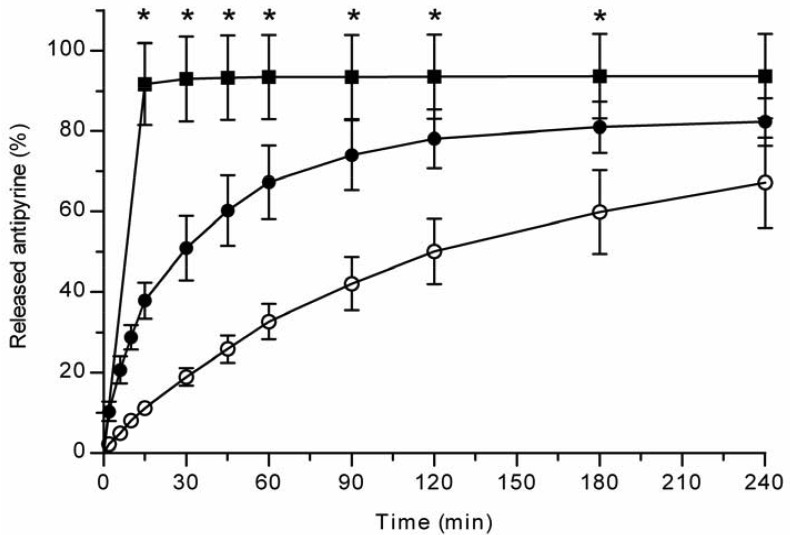
Antipyrine release (%) from TCPSi microparticles at pH 7.4 PBS (+37°C) with centrifuge (■) and well-plate methods (polyester (○) and polycarbonate (•) membranes). Mean ± SD values are shown (n = 3). Statistical differences: * p < 0.05 centrifuge *vs.* well-plate (polyester membrane).

**Figure 3.**
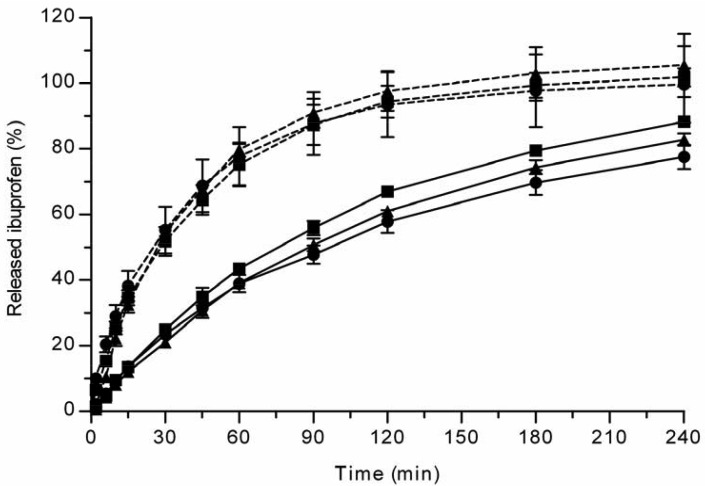
Ibuprofen release from TCPSi microparticles (•), or the transport of ibuprofen applied either as a powder (▲) or a solution (■) into the acceptor chamber across the polyester (solid line) or polycarbonate (dashed line) membranes using the well-plate method. Mean ± SD values are shown (n = 3).

**Figure 4.**
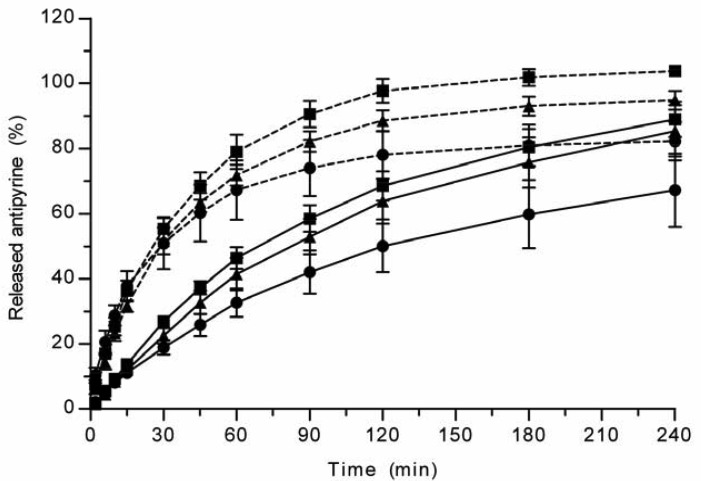
Antipyrine release from TCPSi microparticles (•), or the transport of antipyrine applied either as a powder (▲) or a solution (■) into the acceptor chamber across the polyester (solid line) or polycarbonate (dashed line) membranes using the well-plate method. Mean ± SD values are shown (n = 3).

All the methods showed a fast and complete or nearly complete release of both model compounds from the TCPSi microparticles indicating that these methods were describing *in vitro* drug release equally. Both ibuprofen (Figure 1) and antipyrine (Figure 2) were released within 15 minutes in the centrifuge and USP Apparatus 1 (basket) methods under sink conditions. In the well plate method, the contributions of the actual drug release from the microparticles and the resistance of the membrane to the drug transport rate were evaluated by applying the drugs either as a powder or a solution into the donor chamber. Figure 3 (ibuprofen) and Figure 4(antipyrine) illustrate that the amounts of drug in the acceptor chamber as a function of time display similar shapes irrespective of whether the drug was applied as a solution, a powder or loaded into the TCPSi microparticles. These results indicate that the diffusion of the drugs through the studied membranes was the factor limiting the measured drug release from the microparticles (Figure 1,Figure 2). The barrier role of the membranes is further illustrated by the fact that the difference in the porosity between polycarbonate (12.5%) and polyester (0.5%) membranes clearly affected the rate of drug transport (Figure 3 and Figure 4). Thus, the drug release was rapid also in the well-plate method, and these conclusions were observed under both sink (antipyrine) and non-sink (ibuprofen) conditions.

### 3.2. Ghrelin antagonist release from THCPSi microparticles

The *in vitro* release method needs to be able to incorporate factors such as the suspendability of hydrophobic THCPSi microparticles and the stability of the released peptide. Based on the results described above (Figure 1-Figure 4), the centrifuge method was the technique of choice for evaluating GhA release from hydrophobic THCPSi microparticles. The drug release from porous silicon microparticles is based on diffusion of drug from pores as PSi is wetted [9]. Therefore, it is postulated that the method comparison performed with hydrophilic PSi particles is also valid for hydrophobic PSi as long as the method allows the sufficient wetting of particles. Both USP Apparatus 1 (basket) and centrifuge methods allow an efficient dispersion and wetting of microparticles in the release medium. However, the centrifuge method is more cost-effective as it requires significantly less sample than the USP Apparatus 1 (basket) method. Further, in the centrifuge method, the whole sample volume can be changed at the sampling times, which is beneficial if the drug is rapidly degrading or poorly soluble in the release medium (maintenance of sink conditions).

**Figure 5.**
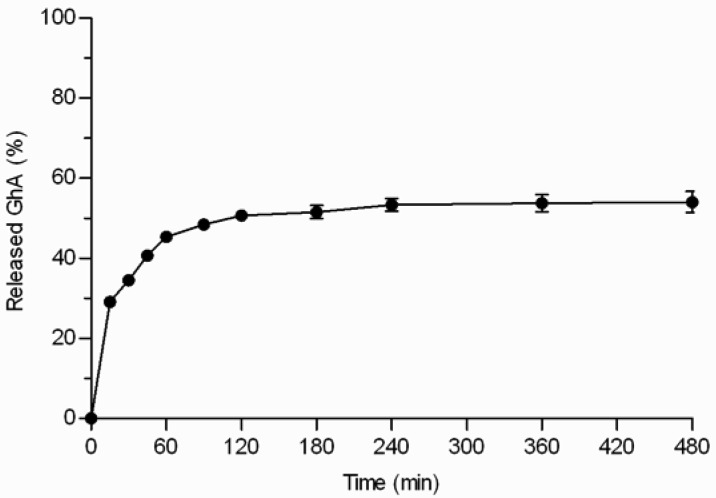
Ghrelin antagonist release from THCPSi microparticles as assessed by the centrifuge method. Mean ± SD values are shown (n = 3).

GhA loaded THCPSi microparticles (loading degree 7.8% w/w) displayed three phases: 1) burst phase; 2) diffusional release phase; and 3) plateau phase (Figure 5). The initial burst release of GhA (29%) occurred within the first 15 minutes. The burst release can be explained by the peptide located on the external surface of the particles and near to the pore orifices as well as release of the multilayers of peptides on the pores not in direct contact with the hydrophobic surface [6,17]. After the burst release, the release was sustained for 2–3 h, and the release reached a plateau when 54% of loaded GhA was released. Even the extraction of the microparticles with ACN/H_2_O mixture at the end of the experiment, did not achieve any additional release (Figure 5). This rather stable plateau between 3 and 8 h suggests that the interactions between GhA and THCPSi slowed down the GhA release and impaired its complete release. This kind of incomplete *in vitro* drug release from particulate drug delivery systems has been reported also earlier [18]. The incomplete release might be also explained by adsorption of BSA, present in the *in vitro* release medium to prevent peptide adsorption onto lab ware. In our studies, BSA was shown to adsorb onto the non-porous THCPSi surface (7.5% w/w) by thermogravimetric analyses [19]. Furthermore, earlier publications have reported human serum albumin adsorption onto a PSi surface [20,21]. Therefore, it is postulated that the BSA adsorption could hinder the release of GhA from the THCPSi pores, resulting in incomplete *in vitro* release.

Previously, *in vivo* delivery via THCPSi microparticles has prolonged the effect of the GhA compared with its administration in solution [8]. Here, this finding was confirmed *in vitro* by using the centrifuge method that allows good particle suspendability and the use of small sample amounts. The sustained peptide release from THCPSi microparticles if compared with the fast release of small-molecular drugs from TCPSi microparticles can be attributed to differences in the physicochemical properties of the loaded molecules and PSi materials. For example, the molecular weight of GhA (M_w_ = 930 g/mol) is substantially higher than that of ibuprofen (M_w_ = 206 g/mol) and antipyrine (M_w_ = 188 g/mol), and THCPSi is more hydrophobic than TCPSi.

## 4. Conclusions

Three different *in vitro* dissolution methods (centrifuge, USP Apparatus 1, well-plate) were compared for testing drug release from hydrophilic TCPSi microparticles; all methods produced comparable results. The centrifuge method was the method chosen to evaluate release of a peptide, *i.e.* a ghrelin antagonist, from the hydrophobic THCPSi particles since this method required only small sample amounts and achieved good particle suspendability. Sustained peptide release from the THCPSi microparticles was observed *in vitro* by the centrifuge method, which is in agreement with an earlier *in vivo* study.

## Figures and Tables

**Table 1.** *In vitro* dissolution methods used for different batches of PSi microparticles and characterization results of average pore diameter before drug load (D), pore volume before drug load (V), drug loading degree (w/w %) and amount of adsorbed drug on the external surface of the microparticles.

**Sample**	**Methods**	**D (nm)**	**V (cm^3^/g)**	**Drug w/w %**	**Surface w/w%**
TCPSi + antipyrine	Centrifuge, well-plate	21	2.1	56	2
TCPSi + ibuprofen	Centrifuge, well-plate	21	2.1	47	0
TCPSi + ibuprofen	USP Apparatus 1	23	1.7	31	0
THCPSi + GhA	Centrifuge	8.9	0.93	7.8	N/A
